# Early neural crest induction requires an initial inhibition of Wnt signals

**DOI:** 10.1016/j.ydbio.2012.02.029

**Published:** 2012-05-01

**Authors:** Ben Steventon, Roberto Mayor

**Affiliations:** Department of Cell and Developmental Biology, University College London, Gower Street, London WC1E 6BT, UK

**Keywords:** Neural crest, Neural plate, Neural plate border, Slug, Snail, *hairy2a*, *dlx5*, *Zic3*, *Msx1*, Wnt, BMP

## Abstract

Neural crest (NC) induction is a long process that continues through gastrula and neurula stages. In order to reveal additional stages of NC induction we performed a series of explants where different known inducing tissues were taken along with the prospective NC. Interestingly the dorso-lateral marginal zone (DLMZ) is only able to promote the expression of a subset of neural plate border (NPB) makers without the presence of specific NC markers. We then analysed the temporal requirement for BMP and Wnt signals for the NPB genes *Hairy2a* and *Dlx5*, compared to the expression of neural plate (NP) and NC genes. Although the NP is sensitive to BMP levels at early gastrula stages, *Hairy2a*/*Dlx5* expression is unaffected. Later, the NP becomes insensitive to BMP levels at late gastrulation when NC markers require an inhibition. The NP requires an inhibition of Wnt signals prior to gastrulation, but becomes insensitive during early gastrula stages when *Hairy2a*/*Dlx5* requires an inhibition of Wnt signalling. An increase in Wnt signalling is then important for the switch from NPB to NC at late gastrula stages. In addition to revealing an additional distinct signalling event in NC induction, this work emphasizes the importance of integrating both timing and levels of signalling activity during the patterning of complex tissues such as the vertebrate ectoderm.

## Highlights

► The neural plate border is a distinct step in the NC induction process. ► Neural plate and border markers respond to Wnt and BMP signals at different times. ► The neural plate border requires an inhibition of Wnt signals during gastrulation. ► An increase in Wnt enables the onset of more specific neural plate border markers.

## Introduction

Induction is a process by which an inducing tissue releases a signal that results in a change in the direction of differentiation of the responding tissue (Gurdon, 1987). Recent molecular explanations for many inductive interactions have revealed increasing complexity, with responding tissues receiving multiple signals from a variety of tissues. One example of this is the induction of the neural crest (NC), an embryonic cell population that arises at the neural plate border that later migrates to numerous sites in the embryo. In *Xenopus* embryos, two separate tissue interactions are thought to induce NC cells. The first involves signals from the dorso-lateral marginal zone (DLMZ) (Bonstein et al., 1998; Marchant et al., 1998; Raven and Kloos, 1945). The second involves an interaction between the neural plate (NP) and epidermis (EP) (Mancilla and Mayor, 1996; Moury and Jacobson, 1989; Selleck and Bronner-Fraser, 1995). Several signalling cascades are required for NC induction, including BMP (Glavic et al., 2004b; Marchant et al., 1998; Mayor et al., 1995; Neave et al., 1997; Nguyen et al., 1998; Wilson et al., 1997), Wnt (Bastidas et al., 2004; Deardorff et al., 2001; Garcia-Castro et al., 2002; LaBonne and Bronner-Fraser, 1998; Lekven et al., 2001; Lewis et al., 2004; Saint-Jeannet et al., 1997; Tribulo et al., 2003), FGF (LaBonne and Bronner-Fraser, 1998; Mayor et al., 1995; Mayor et al., 1997; Monsoro-Burq et al., 2003; Monsoro-Burq et al., 2005; Stuhlmiller and García-Castro, 2012), retinoic acid (Begemann et al., 2001; Villanueva et al., 2002) and Notch (Cornell and Eisen, 2000; 2002; 2005; Endo et al., 2002; 2003; Glavic et al., 2004a).

BMP, Wnt, FGF, RA and Notch signals feed into a complex transcriptional network that include neural plate border (NPB) specifiers such as *Hairy2*, *Msx*, *Ap2*, *Dlx*, *Pax*, *Zic* and *c*-*Myc* gene families (Bang et al., 1997; 1999; Holzschuh et al., 2003; Knight et al., 2003; Luo et al., 2001; Luo et al., 2003; Meulemans and Bronner-Fraser, 2002; Nakata et al., 1997; 1998; 2000; Papalopulu and Kintner, 1993; Sato et al., 2005; Suzuki et al., 1997; Wettstein et al., 1997; Woda et al., 2003). This is followed by a second group termed NC specifiers that include *Snail2*, *Snail*, *Sox9*, *Sox10*, *FoxD3*, *Twist* and *Id3* (Aybar et al., 2003; Honoré et al., 2003; Kee and Bronner-Fraser, 2005; Lee et al., 2004; Light et al., 2005; Linker et al., 2000; Pohl and Knochel, 2001; Sasai et al., 2001).

It remains an open question as to how prospective NC cells interpret multiple extracellular signals such as BMP and Wnt. As a starting point, it is important to ask when each of these pathways is required during NC induction. Interestingly, NC cells change in their requirement for BMP signals (Patthey et al., 2008; Steventon et al., 2009). In *Xenopus* embryos, NC cells require Wnt signals together with intermediate levels of BMP at late gastrula stages for the onset of *Snail2* expression. However, the same cells then require high levels of BMP and Wnt signals during neurulation for maintenance of cell fate (Steventon et al., 2009).

To discover additional steps in the NC induction process, we started by isolating the distinct tissue interactions that are known to be required for NC induction. Based on our previous stage 10 fate map for the neural crest (Steventon et al., 2009) we dissected the prospective NC with different known inducing tissues. We find that in the absence of EP, DLMZ and prospective NC conjugates express the NPB markers *Hairy2a*, *Dlx5*, *Msx1 and Zic3* but not *Pax3* or the NC marker *Snail2*. We next developed a stage 11.5 fate map for the NC from which prospective NC could be taken and cultured in vitro. Interestingly, we find that the NPB markers *Hairy2a* and *Dlx5* are specified at this stage. With these assays we were then able to compare the response of *Hairy2a* and *Dlx5* to NP and NC after modulation of BMP and Wnt signals in distinct time windows. Finally all our in vitro conclusions were confirmed in vivo. Together we present a dynamical model of NC induction, wherein the levels of both BMP and Wnt signalling pathways need to be modulated in three successive steps.

## Materials and methods

### *Xenopus* embryos, micromanipulation and whole-mount in situ hybridization

*Xenopus* embryos were obtained as described previously (Gómez-Skarmeta et al., 1998) and staged according to Nieuwkoop and Faber (1967). Dissections and grafts were performed as described by Mancilla and Mayor (1996). For injection and lineage tracing, β-catenin-GR (Domingos et al., 2001) mRNA was co-injected with FLDx (Molecular Probes) using 8–12 nl needles as described in Aybar et al. (2003). Treatment with dexamethasone was performed as described previously (Tribulo et al., 2003). All plasmids were linearised and RNA transcribed as described by Harland and Weintraub (1985), using SP6 or T7 RNA polymerases, and the GTP cap analogue (New England Biolabs). After DNAse treatment, RNA was purified (BD Biosciences) and resuspended in DEPC-water. For in situ hybridisation, antisense digoxigenin or fluorescein labelled RNA probes were used. Specimens were prepared, hybridized and stained using the method of Harland (1991), and NBT/BCIP or BCIP alone was used as substrates for the alkaline phosphatase. The genes analysed were *Snail2* (formerly *Slug*; Mayor et al., 1995); *Hairy2a* (Wettstein et al., 1997); *Sox2* (Kishi et al., 2000); *Dlx5* (Papalopulu and Kintner, 1993); *Dkk1* (Glinka, et al., 1998); *Zic3* (Nakata et al., 1997); *Msx1* (Maeda et al., 1997) and *Keratin* (Jonas et al., 1989).

### DiI injections and construction of fate map

Injections of DiI (Molecular Probes) were performed at stage 10 as described in Linker et al. (2000). Photos were taken immediately and at stages 11.5 and stage 28. Embryos were sectioned at stage 28 and their fate was determined as previously described (Steventon et al., 2009). Each label was then mapped onto a representative stage 11.5 embryo by counting of superficial cell diameters from the blastopore lip.

### Protein and chemical inhibitor treatment

For proteins, heparin acrylic beads (Sigma) were soaked overnight in 40 μg/ml Dkk1 (Calbiochem), 50 μg/ml Noggin (R and D systems) or 20 μg/ml BMP4 (R and D systems) all suspended in 0.1% BSA. Beads were grafted into explants/whole-embryos for entire culture period prior to fixation.

### Luciferase assay

For each sample, 15–20 explants were taken and homogenised immediately in 25 μl 50 mM Tris–HCl (pH 7.5), centrifuged and a further 25 μl Tris was added to the supernatant. The volume was brought up to 250 μl with the reporter lysis buffer provided with a luciferase assay kit (Promega). The samples were then freeze–thawed and the luciferase activity measured as per manufacturer instructions on a single-tube luminometer (Turner BioSystems). Each reading was standardised by protein concentration as determined by absorbance at 280 nm. This was important to control for differences in tissues sizes of each explant type.

## Results

### The DLMZ is able to promote a sub-set of NPB markers from which the epidermis promotes NC

**Fig. 1:**
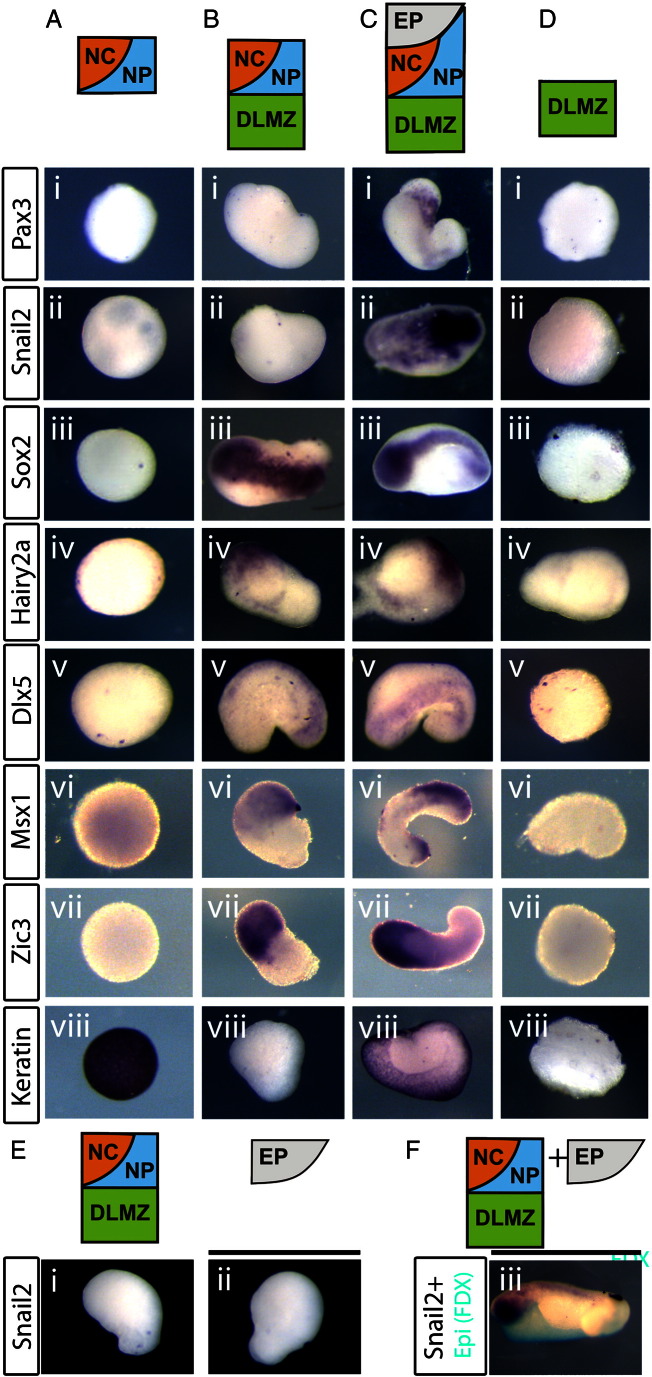
The DLMZ is able to promote expression of neural plate border (NPB) makers in absence of neural crest (NC). Explants of different sizes were taken at stage 10 then cultured for 30 h. A. Prospective neural crest/neural plate (NC/NP) tissue alone, together with dorso-lateral marginal zone (DLMZ; B) or with DLMZ and epidermis (EP; C). D. DLMZ alone. Markers examined: the neural crest markers *Pax3* (i)and *Snail2* (ii), the neural plate marker *Sox2* (iii), the neural plate border markers *Hairy2a* (iv), *Dlx5* (v), *Msx1* (vi) and *Zic3* (vii). Also the epidermal marker *Keratin* (viii). When NC/NP is taken alone, only epidermis is formed (A). In NC/NP/DLMZ explants: only NP and NPB markers are expressed (Biii–viii), but not *Pax3* (Bi) or *Snail2* (Bii). With the addition of the epidermis (EP), all markers are present (C). None of the ectodermal markers tested are expressed in the DLMZ alone (D). E. Explants of NC/NP/DLMZ and FDX-labelled epidermis (EP) were cultured for 30 h either separately or as conjugates. As expected, no *Snail2* expression is observed either in the NC/NP/DLMZ explants (Ei; 0/15) or in the epidermis explants (Eii; 0/15) when cultured alone. However *Snail2* expression (purple) is seen in conjugates (Eiii; 12/15). Epidermis has been stained cyan for FDX label, note that *Snail2* is induced within NC/NP/DLMZ and not in the epidermal region (Eiii).

We aim to discover novel steps in the NC induction process. By isolating specific inducing tissues with prospective NC it is possible to ask whether distinct sub-sets of Neural Plate Border (NPB) markers are expressed prior to the induction of the full complement of NC genes. Although the DLMZ is sufficient to induce NC markers in animal caps (Bonstein et al., 1998; Marchant et al., 1998; Monsoro-Burq et al., 2003; Steventon et al., 2009), it is not known whether this tissue able to promote NC in the absence of prospective epidermis. To test this we explanted the prospective NC with either the DLMZ alone or together with EP. The fate of cells in these regions was known based on our previous stage 10 fate map (Steventon et al., 2009). When explants of the prospective neural crest and neural plate tissue (1A; NC/NP) were cultured in isolation for 30 h, they do not express *Snail2*, *Pax3* (Fig. 1Ai,ii; Table 1), *Sox2* (Fig. 1Aiii; Table 1), *Hairy2a*, *Dlx5* or *Zic3* (Fig. 1Aiv,v,vii; Table 1) markers and are instead reverted to epidermal fate (Fig. 1Aviii, Table 1). A diffuse staining of *Msx1* is observed, which is likely to represent its low level expression in the epidermis (Fig. 1vi; Maeda et al., 1997). When explants of both the prospective NC/NP tissue and DLMZ were cultured for 30 h, specific expression of *Sox2*, *Hairy2a*, *Dlx5*, *Msx1* and *Zic3* markers can be seen (Fig. 1Biii–vii; Table 1) with a reduction in *keratin* expression (Fig. 1Bviii; Table 1). However, these explants still fail to express *Pax3* and *Snail2* (Fig. 1Bi–ii, Table 1) suggesting that further signals might be required for the expression of neural crest markers.

Does the inclusion of EP allow for *Snail2* and *Pax3* expression? Explants of NC/NP together with EP and DLMZ do result in *Snail2* and *Pax3* expression (Fig. 1Ci–ii; Table 1) alongside *Sox2*, *Hairy2a*, *Dlx5*, *Msx1* and *Zic3* (Fig. 1Ciii–vii; Table 1). *Keratin* is also expressed strongly in these explants, confirming the presence of epidermis (Fig. 1Cviii; Table 1). Explants of the DLMZ alone fail to express any of the ectodermal markers examined (Fig. 1D; Table 1). Next we addressed whether the epidermis is releasing signals to induce NC within the NC/NP/DLMZ explants, or whether it is required as additional responding tissue. As expected, neither the NC/NP/DLMZ explants alone (Fig. 1Ei), nor FDX injected-epidermis (Fig. 1Eii) express *Snail2*. When these two tissues are conjugated, a strong expression of *Snail2* in the non-FDX territory is observed, with little or no expression in the epidermis (FDX positive tissue; Fig. 1Eiii). Together, these results suggest that the DLMZ is able to promote the expression of the pan-NPB markers *Hairy2a*, *Dlx5*, *Zic3* and *Msx1* and the NP marker *Sox2*. However, additional signals from the EP are required to induce the posteriorly restricted NPB marker Pax3 and the NC specifier *Snail2*.

### Prospective NC cells are specified to express *Hairy2a*/*Dlx5* at mid-gastrula stages

**Fig. 2:**
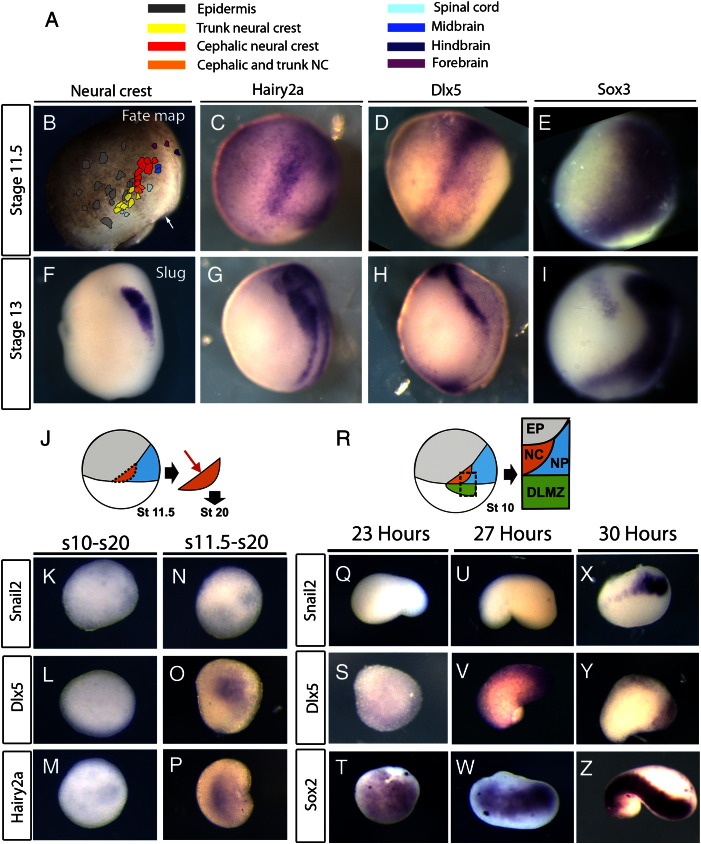
NPB specification occurs earlier than *Snail2*. A, B. Stage 11.5 embryos were injected with small quantities of the lipophilic marker DiI. Groups of cells were marked and their resulting contribution determine at stage 28. The key shows which colours were used in the map to represent each tissue (A). Circles were drawn around each labelled area and mapped onto stage 11.5 (B). Dorsal is to the right, animal to the top. White arrow indicates dorsal blastopore lip. C–E. Expression of *Hairy2a* (C) and *Dlx5* (D) overlaps with the prospective NC, adjacent to the neural plate (NP) marker *Sox3* (E). F–I. At stage 13 the neural crest marker *Snail2* (NC; F) overlaps with the neural plate border (NPB) markers *Hairy2a* (G) and *Dlx5* (H) adjacent to the NP marker Sox3 (I). J–P. Explants of prospective neural crest cells at stage 10 (K–M) or stage 11.5 (N–P) and cultured until sibling embryos were stage 20 and analyzed for *Snail2* (K,N), *Dlx5* (L,O) or *Hairy2a* (M,P). R–Z. Explants of dorso-lateral marginal zone (DLMZ), prospective neural crest and neural plate (NP) and epidermis (EP) were taken together and cultured for various time-points. Expression *Snail2* (Q,U,X), *Dlx5* (S,V,Y) and the neural plate marker *Sox2* (T,W,Z) were analysed by in situ hybridisation. Note that after 23 h only *Sox2* is induced (T), then after 27 h both *Dlx5* and *Sox2* are present (V,W). After 30 h all markers are expressed (X–Z).

What is the specification state of the prospective NC at mid-gastrula stages? To first determine the position of the NC at stage 11.5 we constructed a fate map. Groups of cells were labelled with DiI and their descendants were determined as described previously (Fig. 2A; Steventon et al., 2009). The approximate positions of these labelled cells were mapped onto a representative stage 11.5 embryo by counting cells from the dorsal blastopore lip (white arrow; Fig. 2B). Although the *Snail2* positive neural crest region continues to give rise to some NP and EP derivatives through neurula stages (Linker et al., 2000), this fate map shows that many stage 11.5 clones give rise to predominantly NC derivatives (yellow, orange and red labels; Fig. 2B). The prospective NC region is closely matched with the expression of *Hairy2a*, which is just becoming restricted to this domain at this stage (Fig. 2C). The strongest expression of *Dlx5* also covers the prospective NC territory, although it is also expressed strongly in the anterior neural fold (Fig. 2D). By this time the NP marker *Sox3* is already restricted dorsally to the prospective NP (Fig. 2E). At the end of gastrulation, the specific NC marker *Snail2* is expressed (Fig. 2F) together with *Hairy2a* (Fig. 2G) and *Dlx5* (Fig. 2H), adjacent to the NP (Fig. 2I).

The stage 11.5 fate map allows us to assay the specification state of the NC at mid-gastrulation (Fig. 2J). When the prospective NC/NP region is explanted at stage 10 and grown until sibling embryos are stage 20, neither *Snail2* (Fig. 2K; Table 2) *Dlx5* (Fig. 2L; Table 2) or *Hairy2a* (Fig. 2M; Table 2) is expressed. However, by stage 11.5, *Hairy2a* and *Dlx5* expression is specified (Fig. 2O, P; Table 2) though *Snail2* is not (Fig. 2N; Table 2). Does the NC go through an initial *Hairy2a*/*Dlx5* positive state in response to known inducing tissues? DLMZ, NC/NP and EP explants (Fig. 2R) cultured for 23 h express only the NP marker *Sox2* (Fig. 2Q–T, Table 3). After 27 h both *Dlx5* and *Sox2* are expressed but without *Snail2* (Fig. 2U–W, Table 3). After 30 h *Snail2* is expressed together with *Dlx5* and *Sox2* (Fig. 1X–Z Table 3). Together, these results suggest that the NC is initially specified to express *Hairy2a* and *Dlx5* by the mid-gastrula stage.

### *Hairy2a* and *Dlx5* require low Wnt levels, and are independent of BMP levels

**Fig. 3:**
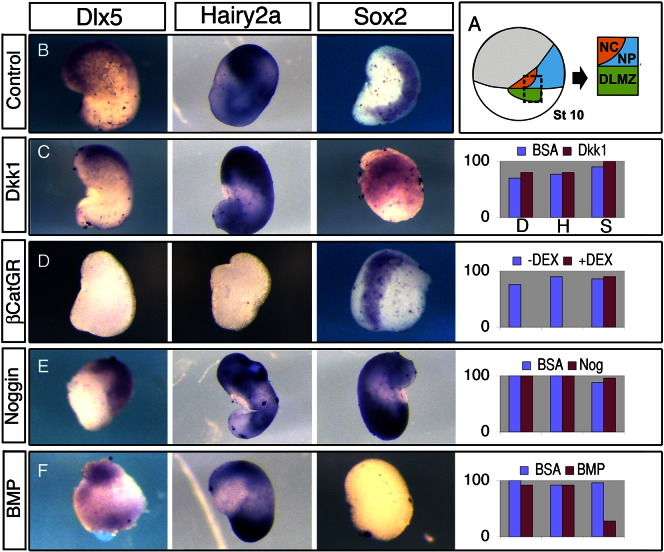
An inhibition of Wnt signalling is required for neural plate border specification. A–F. Explants of prospective neural crest and neural plate with adjacent dorso-lateral mesoderm (NC/NP/DLMZ explants) were taken at stage 10, cultured for 27 h and then analysed for the expression of the neural plate border (NPB) markers *Dlx5* and *Hairy2a* and the neural plate (NP) marker *Sox2*. For each condition the % of explants expressing each marker is shown in the graphs (C–F). B. Control explants cultured with beads soaked in 0.1% BSA (B). C. Explants cultured with Dkk1 protein soaked beads maintained expression of NP and NPB markers. D. Prior to taking explants, embryos were injected at the two-cell stage with β-cateninGR mRNA and explants were cultured in presence or absence (not shown) of dexamethasone. Acitivating β-catenin leads to a loss of NPB border markers, but no change in *Sox2*. E. Explants cultured in the presence of Noggin protein had no affect on NPB markers, and a slight increase in *Sox2*. F. Explants cultured with BMP4 protein had no affect on NPB markers, though *Sox2* was abolished.

The observation that the DLMZ alone is able to promote a subset of NPB markers in the absence of NC enables an assay in which the role of signalling pathways for *Hairy2a* and *Dlx5* expression can be addressed and compared to the later step of NC specification. By taking explants of DLMZ and prospective NC and NP at stage 10, then culturing for 27 h, we can be sure to analyse the expression of *Hairy2a*, *Dlx5* and *Sox2* markers in the absence of *Pax3* and *Snail2* (Fig. 1B,C; Fig. 2R-Z). Dickkopf1 (Dkk1) is an endogenous inhibitor of the canonical Wnt signalling pathway known to be involved in preventing neural crest induction at the anterior neural fold (Carmona-Fontaine et al., 2007; Glinka, et al., 1998). Culture of the explants in the presence of this inhibitor maintained *Hairy2a*/*Dlx5* expression (Fig. 3C) suggesting that an activation of canonical Wnt signals is not required. As a control for Dkk1 activity, we co-cultured NC and epidermal conjugates with beads of Dkk1. With a PBS bead, *Snail2* expression was observed in 95% of cases, while this was reduced to 37% of cases with a Dkk1 bead (data not shown). Interestingly, activation of beta-catenin did result in the inhibition of *Hairy2a*/*Dlx5* although with no effect on *Sox2* expression (Fig. 3D). To address whether levels of BMP signalling are important for the NPB markers *Hairy2a* and *Dlx5*, NC/NP/DLMZ explants were cultured with beads soaked in the BMP inhibitor Noggin (Fig. 3E) and in BMP4 (Fig. 3F). No effect on *Hairy2a*/*Dlx5* is observed in either case, though as expected an inhibition of the neural plate is seen with BMP4 (Fig. 3F).

### The NP and NPB respond to BMP inhibition in successive time-windows

**Fig. 4:**
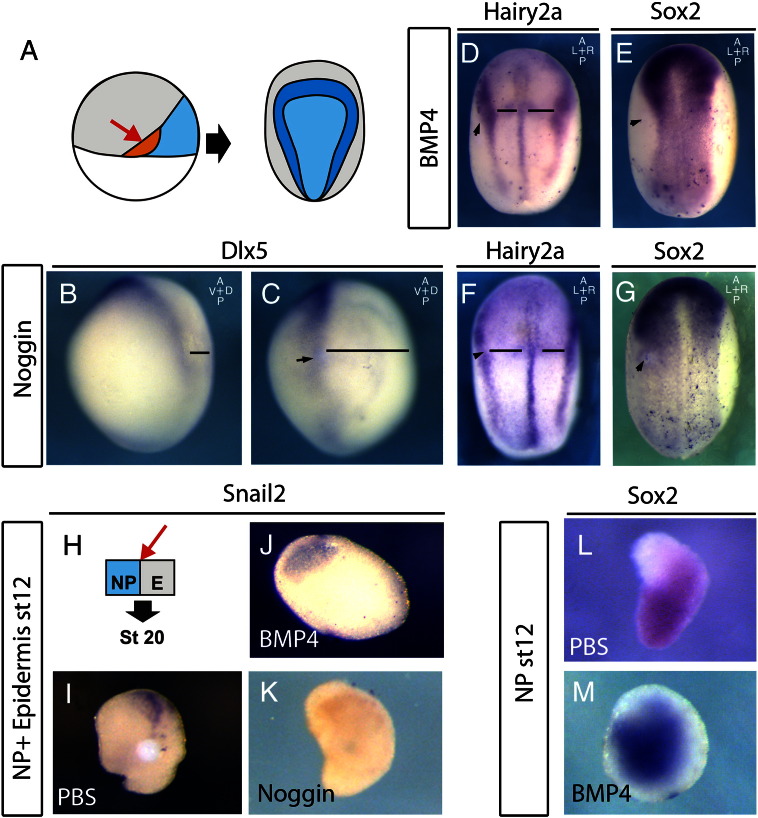
The neural plate (NP) and neural plate border (NPB) respond to BMP signals in successive time windows. A. Experimental design; Embryos were manipulated (red arrow) at the beginning of gastrulation and the affect on neural plate border (*hairy2a*/*dlx5*) and neural plate (*sox2*) markers was assessed. B–C. A bead soaked in Noggin results in lateral shift of *Dlx5* (C) compared to control side (B) when fixed at stage 12. D–E. A bead soaked in BMP4 leads to a shift of *Hairy2a* towards the midline (D; 71%, n = 17) with a corresponding reduction in the Sox2 expression (E; K; 81%, n = 11). F–G. A bead soaked in Noggin leads to a lateral shift in *Hairy2a* but without affecting the thickness of the NPB (F; 69%, n = 13) (F) expression with a corresponding expansion of Sox2 (G; 65%, n = 32 G). H–K. Stage 12 explants of both NP and epidermis (EP) were taken, conjugated and cultured until sibling embryos were at stage 20. I. When co-cultured with a PBS bead, Snail2 expression is induced (70% of cases, n = 20). This is inhibited when co-cultures with either a BMP4 (J; 0% with expression, n = 15) or Noggin (K; 20% with expression, n = 10) soaked bead. L–M stage 12 explants continue to express Sox2 in the presence of either a PBS (L; 100% of cases, n = 7) or BMP4 (M; 100% of cases, n = 8) soaked bead. Black lines indicate distances from midline to the NPB. Black arrowheads indicate position of bead.

The observation that the NP but not NPB markers are affected by modulating BMP levels in our explant assay (Fig. 3E,F) opens the possibility that the NP and NPB respond to BMP inhibition in successive phases. To test this, we grafted beads soaked in Noggin adjacent to the prospective NC at the beginning of gastrulation and assessed the expression of *Dlx5* at stage 12 (Fig. 4A). Interestingly, *Dlx5* was not inhibited by this treatment, but rather shifted ventrally (Fig. 4C) compared to the control side (Fig. 4B). These results suggest that BMP levels are not important for NPB formation during gastrulation, but are important in controlling the size of the neural plate as has been reported in chick embryos (Streit and Stern, 1999). To confirm these results we grafted beads of both BMP4 and Noggin adjacent to the neural plate and analysed the expression of both the NPB marker *Hairy2a* and the NP marker *Sox2* at early neurula stages. BMP inhibition results in an expansion of *Sox2* with no effect on the *Hairy2a* (Fig. 4F, G) and BMP4 leads to an inhibition of *Sox2*, with no effect on *Hairy2a* (Fig. 4D, E).

Subsequent to NPB specification, signals from the EP are required for NC marker expression (Fig. 1D,E). Is this the stage at which the NC is responsive to BMP levels? It is known that at these stages the NC is sensitive to levels of BMP in vivo (Steventon et al., 2009), but we also wanted to confirm this in an explant assay where specific tissue interactions can be isolated. To this end, we conjugated stage 12 NP and EP cultured them until stage 20 together with protein soaked beads (Fig. 4H). PBS beads had no affect on the induction of Snail2 (Fig. 4I). However, *Snail2* expression is lost upon addition of both Noggin and BMP4 (Fig. 4J,K). Interestingly, *Sox2* expression is specified in stage 12 NP explants (Fig. 4L), BMP4 no longer inhibits the NP at this stage (Fig. 4M). Taken together, these results demonstrate that at early gastrula stages the NP is responsive to BMP levels when the NPB is not. However, towards the end of gastrulation, the NC is responsive to BMP levels when the NP is not.

### The NP and NPB respond to Wnt inhibition in successive time-windows

**Fig. 5:**
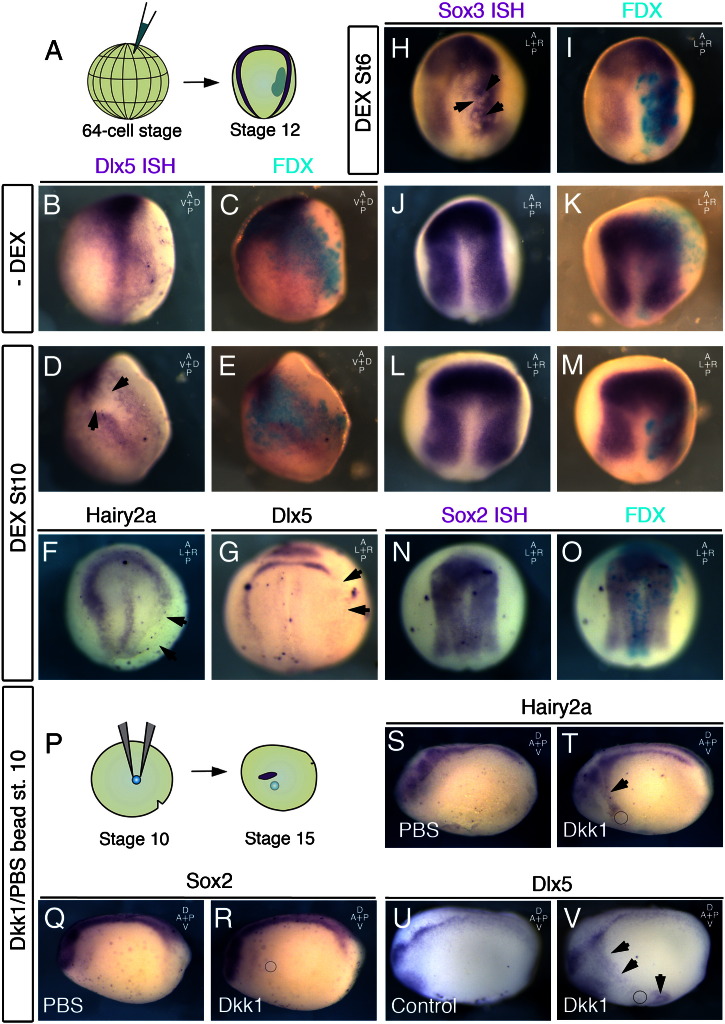
The neural plate (NP) and neural plate border (NPB) respond to Wnt signals in successive time windows. A–M. Embryos were injected at the 64-cell stage with the inducible beta-catenin contruct β-cateninGR and fixed at stage 12 to determine the specific affect on either NP or NPB markers. B–C. No affect on *Dlx5* is seen in the absence of dexamethasone (0% of embryos, n = 9). D–E. *Dlx5* is inhibited within injected cells when the construct is activated at stage 10 (70% with inhibition, n = 10). H–I. Inhibition of Sox2 is observed within injected cells when the construct is activated immediately after injection (75% of embryos with inhibition, n = 16). J–K. Little affect is observed upon Sox3 activation at stage 10 (14% of embryos with slight inhibition, n = 14). L–M. No affect is observed in the absence of dexamethasone 0% of embryos affected, n = 22). F–O. Injection of β-cateninGR at the 32-cell stages leads to a loss of both *Hairy2a* (F; 50% of embryos affected, n = 8) and *Dlx5* (G; 65%, n = 23) at stage 15. No affect was observed in the absence of dexamethasone (data not shown, 0%, n = 24). No inhibition of *Sox2* was observed either in the presence (G; 0%, n = 38) or absence (data not shown, 0% n = 36) or dexamethasone. P–V. Dkk1/PBS or PBS soaked beads were grafted next to the prospective neural crest at stage 10 and embryos were fixed at stage 15. No expansion of the neural plate was detected either with PBS beads (Q; 0%, n = 24) or with Dkk1 (R; 0%, n = 12). No effect was observed with BSA soaked beads on *Hairy2a* (S; 0%, n = 26), an expansion was observed with Dkk1 (T; 63%, n = 36). An expansion of *Dlx5* was also observed with Dkk1 beads (V; 75% of cases, n = 8) compared to control side (U). Black arrows indicate areas affected, black circles indicate position of grafted bead.

Modulating the levels of beta-catenin signalling in our explant assay suggested that an inhibition of canonical Wnt signalling might be required for *Hairy2a*/*Dlx5* expression (Fig. 3C,D). To test this in vivo we injected animal blastomeres at the 64-cell stage with inducible beta-catenin in order to specifically target the NPB or NP in a temporally controlled manner (Fig. 5A). In the absence of dexamethasone *Dlx5* expression appeared normal at stage 12 (Fig. 5B,C). However, activating beta-catenin at stage 10 leads to a loss of *Dlx5* within the injected cells (Fig. 5D,E). Interestingly, activating beta-catenin immediately after injection resulted in a similar reduction of *Sox2* expression in NP targeted cells (Fig. 5H,I). However, this is strongly reduced in the absence of dexamethasone (Fig. 5J,K) or when the construct is activated at stage 10 (Fig. 5L, M). These results are confirmed with 32-cell stage injections with a loss of *Hairy2a* (Fig. 5F) and *Dlx5* (Fig. 5G) seen at stage 13 upon activation at stage 10. The NP marker *Sox2* remains unaffected upon activation of beta-catenin at stage 10 (Fig. 5N, O). What is the effect of reducing canonical Wnt signalling levels during gastrulation? To address this we grafted beads either soaked in PBS alone, or PBS together with Dkk1 (Fig. 5P). Interestingly, no affect on *Sox2* was observed in either case with beads grafted at this stage (Fig. 5Q, R). However, an expansion of both *Dlx5* and *Hairy2a* was observed (Fig. 5T, V) compared to the control situation (Fig. 5 S, U). Our results suggest that although inhibition of Wnt is required for NP induction prior to gastrulation, the NP is unresponsive at gastrula stages. Importantly however, *Hairy2a* and *Dlx5* expression requires an inhibition of Wnt signalling at gastrula stages.

### Increasing levels of Wnt signalling during gastrulation allows for transition from NPB to NC

**Fig. 6:**
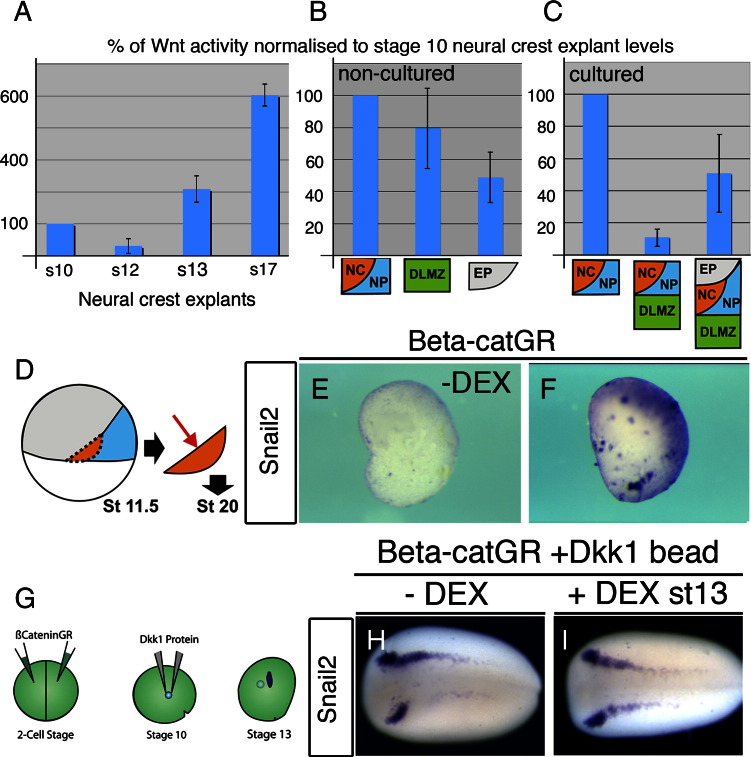
A dynamic modulation of Wnt levels accompanies the transition from neural plate border to neural crest specification. A, B. Measurements of canonical Wnt activity were measured using a TOPflash luciferase reporter assay. Measurements normalised both protein concentration to control for differences in explant size and displayed as a percentage of stage 10 prospective neural crest (NC) activity. Measurements of Wnt activity made from NC explants at different stages (A) or in explants of NC, DLMZ or EP and measured immediately (B). C. Measurements of cultured explants of NC/NP together with either the dorso-lateral marginal zone (DLMZ) or the DLMZ together with epidermis. D–F. Explants of st11.5 prospective NC were taken from embryos previously injected with a dexamethosone (DEX) inducible beta-catenin construct. No *Snail2* expression seen the absence of DEX (0% of cases, n = 21; E), though is expressed upon DEX addition (75% of cases, n = 20; F). G–I. To have an inhibition of Wnt signalling during gastrulation, but a later activation of the signalling pathway during neurulation we first injected 2-cell stage embryos with the inducible construct β-cateninGR. Grafts of Dkk1 soaked beads were then added to the embryos at stage 10, which resulted in an inhibition of Snail2 expression at stage 13 (data not shown, 75%, n = 12). Experimental design (G). In the absence of dexamethasone, this inhibition is still observed at stage 17 (H; 66%, n = 21). However activation of β-cateninGR during the maintenance stage rescues this inhibition (I; 65% of embryos rescued, n = 17). All embryos shown in dorsal view with anterior to the left.

To ask whether levels of Wnt signalling in the NC correlate with the observed changes in requirement for NPB vs. NC, we made use of the TOPflash luciferase reporter of canonical Wnt signalling. NC explants were taken at different stages through gastrulation and neurulation. In order to control for differences in the size of each different explants, and for variation in the efficiency luciferase extraction, each reading was normalised to the total protein concentration of the sample. Luciferase levels are expressed as a percentage of stage 10 NC/NP explant activity and for each sample, each measurement was made in triplicates of 20–25 explants each. Interestingly, a drop in Wnt levels is observed between stages 10 and 12 (Fig. 6A), which correlates with the above observations that Wnt inhibition is required during the specification of the NPB markers *Hairy2a and Dlx5* at these stages. Levels then rise towards the end of gastrulation as NC specifiers are expressed (Fig. 6A). As previously shown, Wnt levels continue to raise during NC maintenance stages (Steventon et al., 2009; Fig. 6A). When stage 10 NC/NP explants or DLMZ explants are taken alone, the level of Wnt signalling is relatively high, compared to levels in the prospective epidermis (Fig. 6B). However, upon culture together, NC/NP/DLMZ explants show a drop in Wnt levels (Fig. 6C), in line with expression of *Hairy2a*/*Dlx5* in absence of *Snail2* and *Pax3* (Fig. 1B). Inclusion of prospective epidermal tissue leads to increased Wnt activity (Fig. 6C), which correlates with the onset of NC marker expression (Fig. 1C). Our results are consistent with the idea that reduced Wnt levels are required for early NPB marker expression, whereas an increase in Wnt activity leads to the onset of more specific NC marker expression.

Is the activation of Wnt signalling sufficient to convert NPB to NC? To test this we took prospective NC from stage 11.5 embryos previously injected with an inducible beta-catenin construct (ßcateninGR; Fig. 6D). These explants are specified to express *Hairy2a* and *Dlx5* but do not express the NC marker *Snail2* (Fig. 1K–M). In the absence of dexamethasone (DEX) beta-catenin is not active, and the explants fail to express *Snail2* (Fig. 6E). However the activation of beta-catenin is sufficient to promote *Snail2* expression (Fig. 6F), supporting the notion that an increase in Wnt signalling in the NPB is sufficient to specify NC markers within the NPB. Taken together, these results suggest that Wnt activation is required for NC only towards the end of gastrulation, to test this we asked whether activation of Wnt signalling at late gastrulation is sufficient to rescue an earlier inhibition of this pathway. Embryos were injected with 125 pg of ßcateninGR mRNA that gave a slight expansion of *Snail2* expression only in a small number of cases (data not shown; 33% of embryos with expansion, n = 12). Subsequently, beads of Dkk1 were grafted adjacent to the prospective NC at stage 10 (Fig. 6G). In the absence of DEX, *Snail2* expression is inhibited (Fig. 6H). Activation of ßcateninGR at the end of gastrulation is able to rescue this (Fig. 6I), suggesting that Wnt activation is required for NC at late gastrula stages.

## Discussion

### A three-step model for NC induction

Here we show that the DLMZ is able to promote the expression of a sub-set of NPB marker expression in the absence of the more specific NPB marker *Pax3* and the NC marker *Snail2*. We also show that further signals from the EP are required for NC induction. In addition, *Hairy2a* and *Dlx5* are both expressed and specified before NC markers. Our results demonstrate the existence of a novel early step in the NC induction cascade that involves the specification of the pan-NPB markers *Hairy2a* and *Dlx5* under conditions of low levels of Wnt signalling. It is known that an increase in Wnt activity within the NPB is required directly for the posteriorly restricted NPB specifiers *Gbx2* and *Pax3* (via *AP2*; Monsoro-Burq et al., 2005; Li et al., 2009; De Crozé et al., 2011). Together, we suggest that pan-NPB markers are specified as a first step under conditions of low Wnt signalling. Subsequently, Wnt activation acts directly on genes such as *AP2* and *Gbx2* to activate the NC transcriptional network within the posterior NPB. These results fit well with the initial suggestions that Wnt signals act as a posteriorising factor in the NPB to induce the NC (LaBonne and Bronner-Fraser, 1998; Villanueva et al., 2002).

**Fig. 7:**
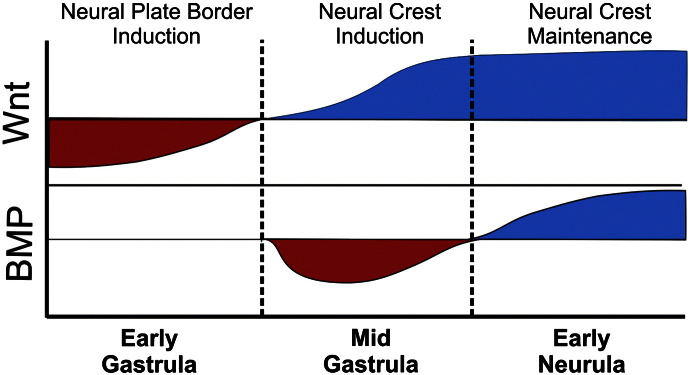
A three-step model for NC induction. Initially Wnt signals need to be repressed for the early neural plate border markers *Hairy2a* and *Dlx5*. Subsequently, an activation of Wnt together with intermediate BMP signals is required for NC specification. Finally, an activation of both BMP and Wnt is required for NC maintenance (see text for details).

We present a three-step model for neural crest induction (Fig. 7). In the first step, a low level of Wnt signalling is required for *Hairy2a* and *Dlx5* expression. FGFs and RA that are expressed in the DLMZ are also likely to be important for this early phase of pan-NPB specification (Monsoro-Burq et al., 2003; Monsoro-Burq et al., 2005; Villanueva et al., 2002). Indeed, blocking FGF signalling with a chemical inhibitor, or with a dominant-negative FGF receptor leads to a loss of *Hairy2a* expression in DLMZ/NC/NP explants (BS and RM unpublished observations). Importantly, the pan-NPB marker *Msx1* has also been shown to be both downstream of FGF signalling (Monsoro-Burq et al., 2005). At this stage, Wnt might act to restrict the expression of *Hairy2a* and *Dlx5* ventrally, as we observe an expansion of these markers in the direction of the non-neural ectoderm only in response to Wnt inhibition. A low level of BMP signalling is most likely to be important to produce a neural plate of the correct size as at these stages these pan-NPB markers are only shifted with changes in BMP levels. Importantly, a very similar result has been observed in chick embryos with beads of Chordin and BMP4 only shifting the expression of the *Msx1* (Streit and Stern, 1999). In addition, a temporal inactivation of BMP signalling in *Xenopus* has been revealed stage specific roles for BMP inhibition in neural and NC induction (Wawersik et al., 2005).

Towards the end of gastrulation the neural crest is evoked. For this an increase in the level of Wnt signalling is required (Bastidas et al., 2004; Deardorff et al., 2001; Garcia-Castro et al., 2002; LaBonne and Bronner-Fraser, 1998; Lekven et al., 2001; Lewis et al., 2004; Patthey et al., 2008; Saint-Jeannet et al., 1997; Steventon et al., 2009; Tribulo et al., 2003). At the same time, neural crest cells are now responsive to an intermediate level of BMP signalling (Glavic et al., 2004a, 2004b; Marchant et al., 1998; Mayor et al., 1995; Neave et al., 1997; Nguyen et al., 1998; Steventon et al., 2009; Wawersik et al., 2005; Wilson et al., 1997). Interestingly, at this time, we find that the neural plate is no longer responsive to BMP levels. Finally, a maintenance signal is necessary from the underlying intermediate mesoderm and involves high levels of Wnts and BMPs (Patthey et al., 2008; Steventon et al., 2009). Here, together with a previous work (Steventon et al., 2009), we have provided embryological assays to distinguish the three steps of NC induction. It would be interesting for future studies to assess the expression of the full complement of NPB and NC specifiers in these assays. This would enable the relation of current models for the NC gene-regulatory network (see Betancur et al., 2010) directly to the tissues and signals that feed into it.

Several observations suggest that ectodermal cells respond at different times to Wnt and BMP signals depending on whether they are fated towards NP or NPB derivatives. It has been demonstrated both in chick and *Xenopus* embryos that the NP border requires an inhibition of Wnt signalling (Heeg-Truesdell and LaBonne, 2006; Wilson et al., 2001). Here we show that the NP is only sensitive to Wnt inhibition prior to gastrulation, when the NPB is sensitive. Importantly, the NP appears to be continually responsive to BMP levels through gastrula stages but become insensitive by stage 12 as specific NC markers are initially expressed. Signalling cascades downstream of BMP, Wnt and FGF signals are thought to converge upon the Smad1 transcription factor (Fuentealba et al., 2007; Pera et al., 2003), and mechanism is thought to be important in regulating the duration of Smad1 activity (Fuentealba et al., 2007). Further experiments monitoring the dynamics of both signalling activity and response will be important in understanding how multiple signals integrated over time and lead to the specification of different cell types such at the NP and NPB derivatives.

### Modulating Wnt levels during gastrulation and neurulation

How the levels of Wnt signalling are modulated such that prospective neural crest cells receive initially a low level during NPB specification, followed by higher levels for NC specification? Although high levels of Wnt signalling are important for Brachyury expression within the marginal zone (Vonica and Gumbiner, 2002), at the beginning of gastrulation, Wnt inhibitors such as Dkk, Frzb, Cereburus and Crescent (Bouwmeester et al., 1996; De Robertis and Kuroda, 2004; Glinka et al., 1998; Leyns et al., 1997; Piccolo et al., 1999; Piccolo et al., 1999; Shibata et al., 2000; Wang et al., 1997) start to be expressed in the organiser, and concomitantly, Wnt8 starts to be downregulated in this territory as a consequence of goosecoid expression (Christian and Moon, 1993; Yao and Kessler, 2001). The DLMZ explants contain a small region of the organiser (Raven and Kloos, 1945; Steventon et al., 2009), likely explaining the low levels of Wnt activity observed in our luciferase reporter assay. *Wnt8* is thought to be important for NC induction in *Xenopus* (Bang et al., 1999; LaBonne and Bronner-Fraser, 1998) and morpholino knockdown resulted in an inhibition of neural crest markers in zebrafish (Lewis et al., 2004). Towards the mid of gastrulation when NC induction starts, *Wnt8* is expressed in the dorsal and lateral mesoderm, closer to the prospective NC and the expression of the Wnt inhibitors moves more anteriorly into the perspective head mesoderm (Carmona-Fontaine et al., 2007; Christian and Moon, 1993; De Robertis and Kuroda, 2004; Glinka et al., 1998; Piccolo et al., 1999). *Wnt10a* is expressed in the epidermis at early neurula stages (Garriock et al., 2007) and might be responsible for the epidermal dependant Wnt activation reported here (Fig. 5A). In chicks, *Wnt6* is expressed in the epidermis (García-Castro et al., 2002). Finally, during neurulation, derivatives of the DLMZ move to underlie the neural crest where a further release of Wnt signals contributes to neural crest maintenance (Steventon et al., 2009).

*Gbx*-*2* and *AP2a* are likely to be two of the earliest transcriptional read-outs of Wnt activation (De Crozé et al., 2011; Li et al., 2009). Loss of AP2a also leads to a loss of *Hairy2a* expression (De Crozé et al., 2011), which might explain the apparent contradiction between the observation that Wnt signalling activity is required for *Hairy2a* expression (Nichane et al., 2008a) and our results presented here, as loss of *Hairy2a* after Wnt inhibition might be a secondary consequence of loosing AP2a expression. In addition to an increase in Wnt signalling, an additional mechanism operates downstream of FGF signalling to mediate the transition from NPB to NC. *Hairy2a* has been shown to be downstream of both BMP and FGF, but not Wnt, activity in *Xenopus* embryos (Nichane et al., 2008a) and is thought to be important for maintaining cells in an undifferentiated state (Guentchev and McKay, 2006; Nagatomo and Hashimoto, 2007; Nichane et al., 2008a; 2008b). Later, via a non-DNA binding dependant mechanism involving Delta and Stat3, *Hairy2a* promotes *Id3* expression. *Id3* is important in promoting NC proliferation and differentiation (Kee and Bronner-Fraser, 2005; Light et al., 2005; Nichane et al., 2008b; 2010). Stat3 is an effector of the FGF signalling pathway, and its levels directly determine the transition between *Hairy2a* mediate NPB proliferation and *Id3* mediated NC specification and differentiation (Nichane et al., 2010). This switch from high to low Stat3 is partly mediated by an *Id3* dependent repression of both *Hairy2a* and the FGFR4/Stat3 complex (Nichane et al., 2010).

What mediates the change in requirement for Wnt signals in the transition from NPB to NC specification, and for BMPs in the transition between NC specification and maintenance? One possibility is that these pathways are required to be kept low in prospective NC cells during specific time windows to avoid unwanted cross-talk with other signalling pathways. FGF and retinoic acid signalling are also known to be essential for NC induction (Begemann et al., 2001; LaBonne and Bronner-Fraser, 1998; Mayor et al., 1995; Mayor et al., 1997; Monsoro-Burq et al., 2003; Monsoro-Burq et al., 2005; Villanueva et al., 2002). It is known that during neural induction, an activation of the MAPK pathway results in the phosphorylation and inhibition of Smad1, a downstream effector of TGFß (Pera et al., 2003). In addition, further interaction has been demonstrated between the BMP-smad pathway and Wnt/Ca^2 +^, the TGF-ß/activin, and the JAK–STAT pathways (for a review see von Bubnoff and Cho, 2001). A complex interaction between Wnt, FGF and retinoic acid pathways is also observed in both chick (Diez del Corral and Storey, 2004; Diez del Corral et al., 2003; Olivera-Martinez and Storey, 2007) and frog embryos (Shiotsugu et al., 2004) during anterior–posterior patterning of the neural axis. As a first step to unravelling some of this complexity it is essential to first analyse the temporal requirement for FGF and Retinoic acid signals in the successive steps of NC induction: NPB specification, NC specification and maintenance. Importantly, it has been shown in *Xenopus* embryos that FGF acts by regulating the levels of Wnt8 in the mesoderm during the initial step neural crest induction, at the mid gastrula stage (Hong et al., 2008).

## Figures and Tables

**Table 1:** Number of different sized explants expressing markers after culture for 30 h.

Marker	NC/NP	NC/NP/DLMZ	NC/NP/DLMZ/EP	DLMZ
*Snail2*	0/31	0/31	21/25	0/16
*Pax3*	0/4	0/19	16/16	0/6
*Sox2*	0/8	23/23	12/12	0/6
*Hairy2a*	0/9	20/20	9/9	0/6
*Dlx5*	0/17	11/11	12/14	0/8
*Zic3*	0/12	6/6	12/12	0/3
*Msx1*	3/6	5/5	16/16	0/9
*Keratin*	6/6	0/17	13/13	0/7

**Table 2:** Number of prospective neural crest explants expressing markers after culture in isolation.

Marker	NC s10-s20	NC s11.5-s20
*Snail2*	0/15	0/17
*Dlx5*	0/12	16/17
*Hairy2a*	0/9	9/11

**Table 3:** Number of NC/NP/DLMZ/EP explants expressing each marker after different periods of incubation.

Marker	NC/NP/DLMZ/EP 23 h	NC/NP/DLMZ/EP 27 h	NC/NP/DLMZ/EP 30 h
*Snail2*	0/24	0/20	18/19
*Dlx5*	0/11	12/13	16/16
*Sox2*	14/14	12/12	21/21
